# Social control and self-control: factors linking exposure to domestic violence and adolescents’ Internet gaming addiction

**DOI:** 10.3389/fpsyt.2023.1245563

**Published:** 2023-08-23

**Authors:** Li Quancai, Cui Meng, Cui Kunjie

**Affiliations:** ^1^Institute for Social Policy and Social Work, Research Institute of Social Development, Southwestern University of Finance and Economics, Chengdu, China; ^2^Department of Sociology, School of Sociology and Psychology, Central University of Finance and Economics, Beijing, China

**Keywords:** exposure to domestic violence, Internet gaming addiction, social control, self-control, parental attachment

## Abstract

Although many studies have investigated the influencing factors of adolescents’ Internet gaming addiction, few have investigated the influence factor of exposure to domestic violence, and even fewer have used the General Strain Theory to explain the influence path of exposure to domestic violence on adolescents’ Internet gaming addiction. Based on the GST, this study sought to uncover further insights into the effect of exposure to family violence on adolescents’ Internet gaming addiction, and the mediating role of social control—specifically, parental attachment—and self-control in the association between exposure to family violence and adolescents’ Internet gaming addiction. Adopting a multi-stage cluster random sampling method, we conducted this study with 2,110 adolescents from Liangshan Yi Autonomous Prefecture in Sichuan Province, China. The results suggest that adolescents’ exposure to domestic violence directly affects their addiction to Internet games and indirectly affects it by decreasing social control and self-control. The study not only supplements and improves the explanatory framework of General Strain Theory, but makes a significant contribution to research on the causes of Internet gaming addiction.

## 1. Introduction

According to the 51st Statistical Report on the Development of Internet in China released by the China Internet Network Information Center, as of December 2022, the number of Internet users in China has reached 1.067 billion, an increase of 35.49 million over December 2021, and the Internet penetration rate has reached 75.6% (1). With the rapid popularization of the Internet, the problem of Internet gaming addiction among adolescents has attracted increasing attention. Internet gaming addiction, an important type of Internet addiction (2), involves playing Internet games in uncontrollable, excessive, or forceful ways that result in physical, psychological, and social dysfunction (3). Researchers commonly recognize individuals as demonstrating Internet gaming addictions when they continue to engage in Internet games even though they are aware of the negative effects of their behaviors (4). Internet gaming addiction among adolescents has also received widespread attention because it is often caused by anxiety, depression, impaired interpersonal relationships, declining academic performance, increased risk behaviors, violent crime, and suicidal behavior (5). Numerous studies have shown that Chinese adolescents demonstrate higher levels of Internet gaming addiction (2.2–21.5%) than their peers in places such as Europe (1.4–9.4%) and the United States (7.6–9.9%) (6–8). Therefore, there is an urgent need to explore the influencing factors of Internet gaming addiction among Chinese adolescents.

The study of children exposed to intimate partner violence (IPV) has accumulated nearly 50 years of research, commencing with the publication of the initial case study in 1975, which provided a comprehensive account of the adverse effects of IPV exposure on children. Then, the primary focus of the research was to identify symptoms in children who were exposed to intimate partner violence (IPV) (9). The findings from the initial cross-sectional studies unequivocally established a robust link between IPV exposure and heightened susceptibility to behavioral, emotional, social, and cognitive difficulties among children (10). Recently, a study using a nationally representative sample of adolescents in China revealed that those who experienced child abuse and/or intimate partner violence (IPV) were considerably more prone to substance misuse, involvement in gambling activities compared to their non-exposed peers (11). However, in the era of widespread Internet access, the relationship between exposure to domestic and Internet gaming addiction remains to be further explored.

In China, the family is generally regarded as the most basic social unit, the most fundamental environment, and the individual’s first experiences of social life (12, 13). Parents’ words and behaviors and family relationships deeply influence adolescent development (12, 13). At present, many studies have explored the relationship between family factors and adolescents’ Internet gaming addiction (14, 15), but only a few studies have specifically explored how exposure to domestic violence impacts Internet gaming addiction. All these studies, without exception, indicate a positive correlation between experiences of domestic violence or exposure to domestic violence and adolescents’ Internet gaming addiction (16–20).

However, no studies have yet examined the mechanisms or pathways through which exposure to domestic violence affects Internet gaming addiction. In this regard, General Strain Theory(GST)can provide insights useful for understanding this process. The concept of strain encompasses three types: 1) the deprivation of positively valued stimuli, 2) exposure to negative stimuli, and 3) the inability to attain desired objectives. These strains are believed to induce negative emotions, spanning from anger to depression and anxiety, which in turn drive individuals to seek ways to relieve the associated negative emotions and/or strains (21–23). Agnew points out that even under the same pressure, many people do not choose deviant behavior (21). This is because the tendency to engage in deviant behavior depends on various factors, including personal relationships with parents and friends and personal psychological factors and personality traits (e.g., self-control and self-restraint). Generally, these prerequisite social factors for deviant behavior comprise factors related to the relationship between individuals and society and factors related to the relationship between individuals and themselves. In other words, the presence or absence of these factors can strengthen or weaken the effect of strain on crime. According to the GST, witnessing parental violence can be a strain for adolescents. The likelihood that an adolescent under this pressure will develop an Internet gaming addiction depends on their relationship with society (social control) and their relationship with their self (self-control).

However, as an adverse life event, the mediating factors linking exposure to domestic violence to the development of Internet gaming addiction, particularly self-control, have not been thoroughly examined. A study in South Korea examined the relationship between domestic violence exposure and Internet gaming addiction as well as the mediating role of social control (parental attachment) (24). However, this study was limited in that it only studied the influence of social control factors (parental attachment) on Internet gaming addiction and not the influence of self-control factors.

Another issue to consider is that despite controlling for negative emotions, the link between strain and crime often persists (25, 26). Additionally, certain studies have found limited or no statistical association between negative affect and criminal behavior (27, 28). Therefore, strain may have a direct effect on criminal behavior. Meanwhile, compensation theory notably suggests that unfulfilled satisfaction in one domain can be compensated by satisfaction in another domain (29). Adolescents who experience or witness domestic violence in long-term family life are likely to experience insecurity and emotional disorders. Considering that adolescents are often in a disadvantaged and powerless position in the family system, they are more likely to engage in Internet games to easily experience control and satisfaction; however, this can lead to Internet gaming addiction. Along these lines, several empirical studies have shown that adolescents who are exposed to aggressive dialog and, relatedly, hostility between parents are more likely to become addicted to Internet games (16–18). For example, Zhao’s study suggests that adolescents exposed to domestic violence have a higher risk of Internet gaming addiction compared to other adolescents, and the more an adolescent experiences domestic violence, the higher their risk of developing an Internet gaming addiction (19). In addition, this study also showed that exposure to domestic violence directly increases Internet gaming addiction (20).

However, previous studies on this topic have mostly focused on adolescents from South Korea; investigations of Chinese samples, especially those from underdeveloped ethnic minority areas, are lacking. Liangshan Prefecture is the largest settlement area for the Yi ethnic group in China, with a total population of 5.3825 million, among which the Yi people account for 54.56%. It is the region with the highest ethnic diversity and the largest population of ethnic minorities in Sichuan Province, China (30). A study conducted in Yunnan Province, China, revealed a higher prevalence of severe alcohol consumption in Yi families compared to Han families (31). Excessive alcohol consumption has been associated with a higher likelihood of domestic violence against women (32). Furthermore, research indicates that domestic violence is prevalent in Yi families in Liangshan Prefecture, with women often bearing the brunt of such violence, leading to a higher probability of children being exposed to domestic violence compared to other regions (33). Historically, Liangshan Prefecture has been a typical case of severe regional poverty in China, characterized by a large impoverished population, deep-rooted poverty, and complex poverty factors (30). Due to relative economic deprivation, a considerable number of parents in Liangshan Prefecture choose long-term labor migration, resulting in a significant population of left-behind children in rural areas. These left-behind children often lack appropriate parental supervision. Combined with the shortage of qualified teachers, low efficiency of family education, and limited school-home collaboration, children in Liangshan Prefecture are more susceptible to Internet gaming addiction (34, 35).

In response, this study examined the influence of exposure to domestic violence on adolescents’ Internet gaming addiction through samples from ethnic minority areas in China and considered the mediating roles of social control and self-control to verify whether GST applies to adolescents’ Internet gaming addiction.

Before further detailing the study, it is necessary to first establish why social control and self-control are important elements to consider when exploring the associations between exposure to domestic violence and adolescents’ Internet gaming addiction. As discussed above, GST suggests that good social control can inhibit deviant behavior under strain; accordingly, social control is a technique and strategy to prevent deviant behavior (36). Specifically, social control refers to a mechanism that regulates individual and group behavior to make people comply with the rules of a specific social group (37). Hirschi, the leading voice of social control theory, believes that social control mainly consists of four concepts: attachment, commitment, involvement, and belief. Among these, “attachment” refers to the degree of dependence and intimacy between individual and social network members (e.g., parents, partners, relatives, friends), which plays the most crucial role in the process of restraining individual bad behaviors (38).

The attachment paradigm may explain the relationship between early exposure to parental violence and parental attachment (39, 40). Many studies have reported data supporting the possible destructive impact of childhood exposure to parental violence on parental attachment. For example, in a study by Sausa et al. (41), adolescents exposed to domestic violence during childhood felt more alienated from their parents than other adolescents. Children exposed to parental violence and neglect are more likely to develop insecure attachment styles than non-exposed children (42, 43). Research by Markiewicz shows that ambivalent perceptions of parents and restless attachment present a static correlation and that witnessing hostility between parents can damage the stability of attachment (44). Unstable attachments to parents are also related to adolescents’ addiction to Internet games. Seob showed that if adolescents’ parents have poor relationships or they have experienced domestic violence, they tend to spend more time using the Internet and smartphones and have a higher risk of becoming addicted to Internet games (45). A series of studies in South Korea have shown that the lower the stability of attachment to parents, the higher the risk of Internet addiction and gaming addiction among adolescents (46–48). These existing studies suggest that parental attachment plays a mediating role in the relationship between exposure to domestic violence and adolescents’ Internet gaming addiction. This also has been explored in previous studies. For example, Dukanac et al. (49) found that exposure to domestic violence indirectly affected addiction through a decrease in parental attachment.

Meanwhile, self-control is an important psychological function by which individuals actively control unreasonable thoughts, emotions, and behaviors to conform to social norms and achieve long-term goals (50, 51). Previous studies have found that individuals with high levels of self-control can better control and suppress negative behavioral responses. Conversely, a lack of self-control can affect an individual’s executive control function, leading to irrational decision-making (52, 53). Agnew stated that self-control may be the most potent factor affecting the relationship between strain and aggressive behavior (22). The formation of self-control in adolescents is related to parenting practice and parental self-control. Gottfredson and Hirschi argue that parents with low self-control are likely to be ineffective at parenting and produce children with lower self-control (54). Meldrum et al. (55) provide evidence of an indirect association between maternal self-control and early childhood self-control through maternal ineffective parenting. Considering that exposure to domestic violence implies that parents have a lower level of self-control, which can affect adolescents’ self-control, we can infer that exposure to domestic violence is a predictive factor for adolescent self-control.

For our purposes, it is important to remember that a lack of self-control is closely related to Internet gaming addiction. Multiple studies have shown a significant relationship between self-control and Internet gaming addiction. The weaker the individual’s self-control, the more likely they are to develop an Internet addiction (56–59). Faced with the stimulation and temptation of the virtual network world, individuals with high levels of self-control can rationally regulate their network use and will not overindulge in the pleasure brought by the network. In contrast, individuals with low levels of self-control will find it difficult to extricate themselves from the virtual world, and even use it to escape real-life problems (60). Therefore, considering the theory of self-control and empirical studies on the relationship between domestic violence exposure, self-control, and Internet game addiction, we propose that self-control may play a mediating role between exposure to domestic violence and adolescent addiction to Internet games.

Based on the overall framework of GST, this study examined the impact and pathway of exposure to domestic violence (strain) on Internet gaming addiction (deviant behavior). Unlike previous studies, we proposed that exposure to domestic violence leads to adolescent Internet gaming addiction not only by reducing social control (parental attachment), but also by weakening self-control. With reference to the literature, we proposed the following hypotheses:

1. Exposure to domestic violence positively predicts adolescents’ Internet gaming addiction.
2. Exposure to domestic violence positively predicts adolescents’ Internet gaming addiction through the mediating role of social control.
3. Exposure to domestic violence positively predicts adolescents’ Internet gaming addiction through the mediating role of self-control.

## 2. Method

### 2.1. Participants

Adopting a multi-stage cluster random sampling method, this study recruited 2,110 adolescents (grades 7–8) attending five secondary schools in Liangshan Yi Autonomous Prefecture, Sichuan Province, China. As shown in Table 1, there were 987 boys and 1,123 girls in the sample, with an average age of 14.70 years old (range 7–18 years). The family economic status of most participants was low- or middle-income, and many of the participants’ parents did not attend or finish primary school. The study was approved by our university’s research ethics committee, and all respondents were informed of their rights of informed consent, voluntary participation, anonymity, and confidentiality. In the analysis, multiple imputations were adopted to handle missing values.

**Table 1:** Descriptive statistics of the participants.

	Frequency (*n*)	Percentage (%)
Gender		
Boy	987	46.8
Girl	1,123	53.2
Age	*M* = 14.70 (years)	Range = 7–18 (years)
Grade		
Grade 7	1,141	54.1
Grade 8	969	45.9
Family economic status		
Very poor	242	11.5
Poor	962	45.6
Middle-class	886	42.0
Wealthy	15	0.7
Very wealthy	5	0.2
Father’s/**mother’s** highest level of education		
Did not attend or finish primary school	1040/**1522**	49.3/**72.1**
Primary school	730/**403**	34.6/**19.1**
Secondary school	273/**146**	12.9/**7.0**
High school	43/**14**	2.1/**0.6**
Vocational college	15/**20**	0.7/**1.0**
Junior college	8/**4**	0.4/**0.2**
Bachelor’s degree or higher	1/**1**	0.0/**0.0**

### 2.2. Measures

*Exposure to domestic violence* was measured with three items assessing how often adolescents witness conflict between their parents. Each item was rated on a 5-point Likert scale ranging from 1 (*never*) to 5 (*always*), with higher scores representing more exposure to domestic violence. Representative items included “I have witnessed my family being loudly scolded, insulted, or humiliated by other family members” and “I have witnessed my family being slapped, kicked, punched or beaten by other family members.” The reliability of the scale was found to be acceptable for the participants in this study (Cronbach’s *α* = 0.66).

*Social control* was measured by the Parental Attachment subscale taken from a revised version of Hirschi’s Bonding Scale (61). This subscale contains four items, such as “I talk over future plans with my parents” and “I share my thoughts and feelings with my parents.” Participants responded to each item on a 5-point scale ranging from 1 (*strongly disagree*) to 5 (*strongly agree*). In analysis, the four items were reverse coded, with higher scores representing less social control from parents. In our study, Cronbach’s alpha for the instrument was 0.72.

*Self-control* was measured by the Temper subscale taken from Grasmick et al.’s Self-control Scale (62). This subscale contains four items following a 4-point Likert-type scale, ranging from *strongly disagree* to *strongly agree*. Higher scores on the scale indicate a higher likelihood of becoming angry and lower levels of self-control. Representative items included “I lose my temper easily” and “When I am angry at people, I feel more like hurting them than talking to them about why I am angry.” The original version of the scale has demonstrated good explanatory power in a sample of Chinese adolescents (63), and the Cronbach’s alpha for the Temper subscale in our study was 0.70.

*Internet gaming addiction* was measured by the Internet Gaming Disorder Scale-Short Form (64), which comprises nine items assessing the degree of individuals’ addiction to Internet gaming. All the nine items were rated using a 5-point Likert-type scale (1 = *never* and 5 = *very often*), with a higher score on the scale indicating a higher risk of developing an Internet gaming addiction. Representative items included “Do you systematically fail when trying to control or cease your gaming activity?” and “Do you feel the need to spend increasing amounts of time engaged in gaming in order to achieve satisfaction or pleasure?” The nine-item scale has previously been validated with promising psychometric properties in English, Italian, Turkish, Persian, Portuguese, Albanian, and Chinese samples (65). The Cronbach’s alpha for the scale in our study was 0.87.

*Covariates.* In the analysis, gender (1 = *boy*, 2 = *girl*), age, and family socioeconomic status (SES; assessed by parents’ highest level of education and family economic status) were included as control variables.

### 2.3. Data analysis

Preliminary analyzes, including mean scores, standard deviations, and bivariate correlation, were performed using SPSS 24.0. The measurement model and structural equation modeling were conducted in AMOS 26.0 to test the factor loadings, model fits, and the direct and indirect effects between variables. The indirect effects were also confirmed by performing bootstrapping analyzes with 5,000 resamples and 95% bias-corrected confidence intervals. In addition, multiple indices were used to determine whether the models fit the data well, including Chi-square divided by degrees of freedom (χ^2^/*df*, 3–6 acceptable), the comparative fit index (CFI, >0.90 acceptable), and the root mean square error of approximation (RMSEA, <0.08 acceptable) (66).

## 3. Results

### 3.1. Descriptive and correlations

Mean scores, standard deviations, and bivariate correlations of all variables are presented in Table 2. As shown, the primary variables were significantly correlated with each other in the expected directions. In particular, exposure to domestic violence was positively correlated with social control (*r* = 0.186, *p* < 0.01), self-control (*r* = 0.183, *p* < 0.01), and Internet gaming addiction (*r* = 0.223, *p* < 0.01). Meanwhile, both social control (*r* = 0.196, *p* < 0.01) and self-control (*r* = 0.255, *p* < 0.01) were significantly correlated with Internet gaming addiction. Moreover, the control variables were significantly correlated with some of the primary variables. The direct effect of exposure to domestic violence on Internet gaming addiction was also examined, and the results indicated that exposure to domestic violence was significantly associated with higher levels of Internet gaming addiction among the adolescents in the study (*β* = 0.27, *p* < 0.001).

**Table 2:** Bivariate correlation between variables.

	*M*	*SD*	1	2	3	4	5	6	7
1. Exposure to domestic violence	1.27	0.50	1						
2. Social control	2.43	0.93	0.186^**^	1					
3. Self-control	1.91	0.66	0.183^**^	0.141^**^	1				
4. Internet gaming addiction	1.44	0.61	0.223^**^	0.196^**^	0.255^**^	1			
5. Gender	1.53	0.50	−0.027	−0.017	−0.019	−0.329^**^	1		
6. Age	14.7	1.2	0.059^**^	0.015	−0.002	−0.043^*^	0.045^*^	1	
7. SES	0.69	0.19	−0.070^**^	−0.044^*^	0.001	0.066^**^	−0.126^**^	−0.185^**^	1

^**^ Significant at the 0.01 level (2-tailed).

^*^ Significant at the 0.05 level (2-tailed).

### 3.2. Test of the measurement and structural models

We performed the measurement model with four latent variables. The results showed a good fit to the data: *χ*^2^ = 899.685, df = 164, *χ*^2^/df = 5.486, *p* < 0.001; CFI = 0.943; RMSEA = 0.046; SRMR = 0.035. All the factor loadings for the indicators on the latent variables were significant at the *p* < 0.001 level. The factor loadings of exposure to domestic violence, social control, self-control, and Internet gaming addiction ranged from 0.532–0.777, 0.356–763, 0.525–680, and 0.556–798, respectively. The results indicated that the observed indicators effectively represented their latent variables.

**Figure 1:**
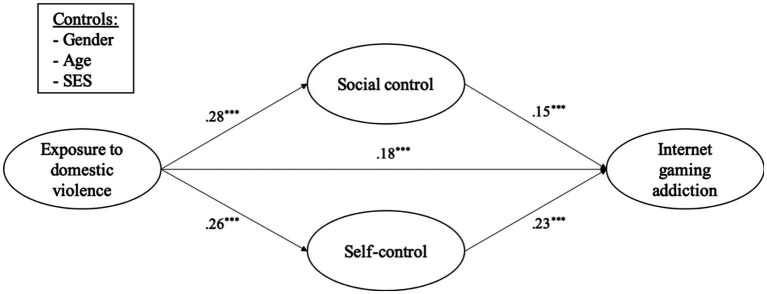
Standardized structural model of the direct and indirect effects. ^***^p < 0.001.

The test of the hypothesized structural model with mediators also showed that the sample provided a good fit to the data (*χ*^2^ = 1252.532, df = 219, *χ*^2^/df = 5.719, *p* < 0.001; CFI = 0.923; RMSEA = 0.047; SRMR = 0.038). A total of 26.6% of the variance in adolescents’ Internet gaming addiction was explained by the model. Figure 1 and Table 3 show the estimates of the structural model and bootstrapping results of the direct and indirect effects. As hypothesized, the results revealed significant indirect effects of exposure to domestic violence on adolescents’ Internet gaming addiction *via* social control (95% CI: [0.044, 0.133], *β* = 0.080, *p* < 0.001). Specifically, exposure to domestic violence was positively associated with social control (*β* = 0.28, *p* < 0.001), which further increased adolescents’ Internet gaming addiction (*β* = 0.15, *p* < 0.001). Exposure to domestic violence also had a significant indirect effect *via* self-control on adolescents’ Internet gaming addiction (95% CI: [0.072, 0.175], *β* = 0.113, *p* < 0.001). Specifically, exposure to domestic violence was positively associated with self-control (*β* = 0.26, *p* < 0.001), which further increased adolescents’ Internet gaming addiction (*β* = 0.23, *p* < 0.001).

**Table 3:** Estimates of the structural model and bootstrapping results of the direct and indirect effects.

Estimates of the structural model	B	*β*	SE	CR	*p*
Exposure to domestic violence ➔ Internet gaming addiction	0.342	0.18	0.055	6.240	***
Exposure to domestic violence ➔ Social control	0.766	0.28	0.086	8.939	***
Exposure to domestic violence ➔ Self-control	0.455	0.26	0.059	7.724	***
Social control ➔ Internet gaming addiction	0.104	0.15	0.017	5.943	***
Self-control ➔ Internet gaming addiction	0.247	0.23	0.029	8.412	***

Bootstrapping results of the direct and indirect effects	β	SE	*p*	95%CI	
				Lower	Upper
Direct effect					
Exposure to domestic violence ➔ Internet gaming addiction	0.180	0.040	0.000	0.104	0.262
Specific indirect effect					
Exposure to domestic violence ➔ Social control ➔ Internet gaming addiction	0.080	0.022	0.000	0.044	0.133
Exposure to domestic violence ➔ Self-control ➔ Internet gaming addiction	0.113	0.026	0.000	0.072	0.175
*Total effects*	0.282	0.035	0.000	0.214	0.355

***p < 0.001.

Apart from the significant indirect effects, exposure to domestic violence also demonstrated a significant direct effect on Internet gaming addiction (95% CI: [0.104, 0.262], *β* = 0.180, *p* < 0.001) in the structural mediation model. This means that social control and self-control partially mediated the relationship between exposure to domestic violence and adolescents’ Internet gaming addiction. Of all the control variables, only gender was significantly associated with Internet gaming addiction (*β* = −0.32, *p* < 0.001), indicating that boys were more likely to be addicted to Internet gaming than girls.

## 4. Discussion

Based on GST, we explored how social control (parental attachment) and self-control mediate the effect of exposure to family violence on adolescents’ Internet gaming addiction by studying a sample of 2,110 adolescents from Sichuan Province, China. Initially, we proposed that the adolescents’ exposure to domestic violence would directly affect their addiction to Internet games and, additionally, indirectly affect their addiction by decreasing their social control and self-control.

### 4.1. Relationship between exposure to domestic violence and addiction to Internet games

The results revealed that exposure to domestic violence positively predicts adolescent Internet gaming addiction. This result is consistent with most previous research, which reports significant and positive relations between exposure to domestic violence and Internet gaming addiction (19–21, 24, 29). These findings have been explained by the social compensation model. Children exposed to parental violence are more likely to have insecure attachments with their parents (42, 43). Positive experiences for adolescents immersed in Internet gaming could compensate for poor parent–child relationships, which can lead to pathological Internet use (67). Meanwhile, the findings of this study demonstrate a direct effect of strain on deviant behavior, providing new insights into the GST. At present, there are few studies on the relationship between exposure to domestic violence and Internet gaming addiction, and most of them feature Korean samples. The current study was based on samples from ethnic minority areas in China and therefore further enriches the relevant empirical research.

### 4.2. The mediating role of social control

The results revealed that exposure to domestic violence had a significant indirect effect on adolescents’ Internet gaming addiction *via* social control. Specifically, exposure to domestic violence was positively associated with social control, which further increased adolescents’ Internet gaming addiction. This result was consistent with the previous research in Korea that found that parental attachment had a mediating effect on the relationship between exposure to domestic violence and Internet gaming addiction (24). Social control theory can be used to explain this result. According to this theory, individuals obey rules because of social control (68)—while there is a potential deviant motivation in everyone’s human nature, individuals do not engage in deviant behavior because of effective social control (37). Therefore, we may consider that while every adolescent is a potential addict, some do not have Internet gaming addictions because they developed connections with multiple subjects, including parents, during their socialization process, which effectively controls their Internet usage behavior.

Interestingly, different findings have emerged regarding the relationship between exposure to domestic violence and parental attachment. For example, an Israeli study suggested that domestic violence had distinct effects on children’s perception of their relationships with their parents that differed depending on whether the children were themselves victims of that violence. Notably, the study reported that witnessing spousal abuse had little impact on children’s perceived relationships with their parents (69). However, a study in South Korea found that parental attachment completely mediates exposure to domestic violence and Internet gaming addiction (24), which reflects the significant utility of parental attachment. The results of this study suggest that children in East Asian countries may be more sensitive to the interaction patterns of their family members. Especially within Yi culture, individuals are not seen as independent entities but rather as integral members of their extended families. Close interaction among family members constitutes the most significant and primary relationships (70). Consequently, Yi children exhibit heightened sensitivity to the quality of family relationships, which may explain how witnessing family violence can undermine their attachment to their parents.

### 4.3. The mediating role of self-control

The results revealed that exposure to domestic violence also had a significant indirect effect *via* self-control on adolescents’ Internet gaming addiction. Specifically, exposure to domestic violence was positively associated with self-control, which further increased adolescents’ Internet gaming addiction. To some extent, this finding is consistent with the results of previous studies. In real life, long-term exposure to domestic violence can cause pain, anxiety, depression, and insecurity, while consuming self-control resources. When the self-control protection system cannot be restored promptly, the ability to practice self-control will decrease (71–73). Compared to adults, adolescents have weaker self-control abilities, so when they are exposed to Internet games, they are prone to exceeding their reasonable usage time, leading to Internet gaming addictions. As Meldrum et al. surmised, children and adolescents with parents with lower levels of self-control and closely related personality traits are more likely to be exposed to less nurturing family environments, to have lower levels of self-control themselves, and to engage in antisocial behavior (55). In Yi society, parental role modeling plays a paramount role in the socialization of children. A plethora of proverbs in Yi daily life expound upon the influence of parents in shaping their children’s behavior patterns, such as “Parents who speak kindly teach children to be courteous,” “When mothers steal salt, daughters will steal chili,” and “Parents behaving uncivilly lead to coarse language in their children” (74). These expressions underscore the significance of family atmosphere in influencing children’s behavioral patterns. Exposure to family violence is perceived as a manifestation of parental lack of self-control, which in turn affects the self-control abilities of the children and consequently increases the likelihood of Internet gaming addiction.

There is limited research on the mediating role of self-control in domestic violence exposure and Internet gaming addiction. This study can guide future research on the role of self-control in regulating strain and deviant behaviors in GST. For example, future research can apply this study’s model to other explanations of deviant behavior. In addition, the results also notably revealed that boys are more likely to become addicted to Internet games than girls, which is consistent with previous studies (75, 76).

## 5. Contributions, limitations, and future directions

This study makes several contributions to existing research and practice. Notably, this study incorporated social control theory and self-control theory into the framework of GST, formed a new explanatory model for deviant behavior, and verified this model. GST explains how experiencing strain leads to deviant behaviors such as crime. This study applied GST to explore how children develop Internet gaming addiction after experiencing exposure to domestic violence. Previous studies on the application of GST only considered the mediating factors of social control in strain and deviant behavior (49, 77). This study expanded the theory’s application scope to Internet gaming addiction. The results show that exposure to domestic violence can predict adolescent Internet gaming addiction through the mediating effects of social control and self-control. These findings evidence that, under strain, adolescents will demonstrate less social control and self-control, which increases their risk of deviant behavior. The study not only supplements and improves the explanatory framework of GST, but makes a significant contribution to research on the causes of Internet gaming addiction.

Additionally, the results of this study provide ideas for practice on how to address adolescent Internet gaming addiction, especially in Yi society. Considering the impact of exposure to domestic violence and parental attachment on adolescents’ addiction to Internet games, the study emphasizes that preventive measures are necessary; for example, parents should be encouraged to avoid conflicts in front of their children and to maintain good relationships with their children. Meanwhile, social work intervention projects with the theme of self-control should be further valued and implemented to prevent and solve the problem of adolescent Internet gaming addiction. Considering the scarcity of professional social services in Yi areas, local governments may contemplate introducing specialized social organizations to implement such practices in order to reduce domestic violence and enhance self-control among adolescents.

This study also has some limitations. First, because a cross-sectional design was employed, no causal inferences were determined. Future studies could use experimental designs to further verify the results of the present study. Second, this study’s sample only included adolescents from poverty-stricken areas in China, which may limit the generalizability of the results; therefore, future studies could use other samples in China or other countries to verify the current results.

## 6. Conclusion

This study offers three conclusions. First, exposure to domestic violence has a significant direct effect on adolescents’ Internet gaming addiction. Second, exposure to domestic violence has a significant indirect effect on adolescents’ Internet gaming addiction *via* social control. Specifically, exposure to domestic violence was found to be positively associated with social control, which further increased adolescents’ Internet gaming addiction. Third, exposure to domestic violence also had a significant indirect effect *via* self-control on adolescents’ Internet gaming addiction. In particular, exposure to domestic violence was positively associated with self-control, which further increased adolescents’ Internet gaming addiction. In addition, the study also found that boys are more likely than girls to develop an Internet gaming addiction.

## Data availability statement

The raw data supporting the conclusions of this article will be made available by the authors, without undue reservation.

## Ethics statement

The studies involving humans were approved by Research Institute of Social Development Southwestern University of Finance and Economics. The studies were conducted in accordance with the local legislation and institutional requirements. Written informed consent for participation in this study was provided by the participants’ legal guardians/next of kin. Written informed consent was obtained from the minor(s)’ legal guardian/next of kin for the publication of any potentially identifiable images or data included in this article.

## Author contributions

LQ, project leader, is responsible for data collection and participating in paper writing. CM is responsible for the primary writing, polishing, revision, and submission of the paper. CK is responsible for data collection, data analysis, and participating in paper writing. All authors contributed to the article and approved the submitted version.

## Funding

This study is supported by the Youth Program of the National Social Science Foundation of China (Project Name: Dynamic Development and Precision Intervention Research on Rural Left behind; Approval Number: 22CSH068); 2023 Central University Basic Research Business Fee Project (Project Name: A Study on the Realistic Dilemma and Implementation Path of Organizational Resilience Cultivation in Social Work Institutions in Ethnic Regions; Approval Number: JBK2304089); Liangshan Prefecture Relocation and Poverty Alleviation Community Governance Service Project.

## Conflict of interest

The authors declare that the research was conducted in the absence of any commercial or financial relationships that could be construed as a potential conflict of interest.

## Publisher’s note

All claims expressed in this article are solely those of the authors and do not necessarily represent those of their affiliated organizations, or those of the publisher, the editors and the reviewers. Any product that may be evaluated in this article, or claim that may be made by its manufacturer, is not guaranteed or endorsed by the publisher.
